# EvidenceToPrograms.com: A Toolkit to Support Evidence-Based Programming for Seniors

**DOI:** 10.3389/fpubh.2015.00018

**Published:** 2015-04-27

**Authors:** Alan B. Stevens, Shannon B. Coleman, Richard McGhee, Marcia G. Ory

**Affiliations:** ^1^Baylor Scott & White Health, Temple, TX, USA; ^2^Central Texas Area Agency on Aging, Belton, TX, USA; ^3^Program on Healthy Aging, School of Public Health, Texas A&M Health Science Center, College Station, TX, USA

**Keywords:** toolkit, evidence-based programming, older adults, community-based organizations, senior citizens

Community-based organizations (CBOs) are moving rapidly to provide new health services proven to be health promoting, and importantly, desired by seniors. In particular, evidence-based programs (EBPs) that promote healthy behaviors and proper self-management of health conditions have become a valuable resource to CBOs by complementing formal healthcare services. Yet, the level of support needed for community adoption and implementation of current EBPs is insufficient to maximize CBOs’ ability to enhance the health of the senior population. Decision-making support is a critical factor in facilitating the use of EBPs such as those listed on national clearinghouses (e.g., National Council on Aging, NCOA) that address a local need or the need of a specific senior population. The Community Research Center for Senior Health (CRC-Senior Health) developed a web-based Toolkit (EvidenceToPrograms.com) to support CBOs in the selection, implementation, and evaluation of EBPs^1^. This Commentary will describe the rationale behind the tool, its development and basic structure and content. Additionally, plans for future development will be shared.

## What is the Purpose of this Toolkit?

The purpose of this Toolkit is to build the capacity of CBOs to promote senior health and well-being through evidence-based programming. The toolkit does not promote or advocate for any specific EBP. Rather, it is intended to guide a user through a series of steps and decisions to facilitate an organization’s ability to provide programming that is desired and beneficial to the seniors being served.

## Who is the Intended Toolkit Audience?

The Toolkit is designed for organizations that are motivated to implement EBPs for seniors. Whether organizations are new to evidence-based programming or have been implementing EBPs for years, they will find useful materials in the Toolkit. For organizations with minimal experience, the Toolkit will function as a guide that provides the basic information needed to select, implement, and evaluate an EBP. For organizations with significant experience, the Toolkit will function as a primer that is useful in evaluating their approaches to program selection, implementation, and evaluation. Several ways are described in which organizations that serve seniors can optimize their use of the Toolkit. The Toolkit is also a useful and user-friendly online resource for healthcare professionals and students interested in learning about evidence-based programming. The Toolkit is designed to be a resource for organizations throughout the US who are interested in the promotion of health, regardless of the organization’s target clientele.

## How was the Toolkit Developed?

Toolkit development spanned 18 months and consisted of five major tasks: (1) determining which topics to address in the Toolkit; (2) scanning existing materials to identify resource gaps and materials to reference in the Toolkit; (3) developing the Toolkit content; (4) refining the Toolkit with adjustments suggested by a team of expert reviewers; and (5) creating a user-friendly website to feature the Toolkit. The diverse, expert advisory panel representing community health and health promotion researchers, state-level aging services, local community-based service providers, and national leaders in evidence-based programming reviewed the Toolkit content and provided feedback during development.

## What is the Format of the Toolkit?

The format was designed based on feedback received during its development. The Toolkit is featured on an interactive, user-friendly website, EvidenceToPrograms.com. Users can explore paths for learning how to select an EBP as well as how to implement a selected program.

## What Content is in the Toolkit?

The Toolkit provides a comprehensive overview of program selection, implementation, and evaluation. The content of the Toolkit is divided into two sections: (1) selecting a suitable EBP and (2) implementing EBPs with fidelity. This includes information not commonly found on EBPs.

Each section is further divided into subsections offering questions, examples, and resources to help organizations anticipate and address barriers to implementing or maintaining a program. For example, Section 1 has three subsections: (a) what does it mean for a program to be evidence-based; (b) choosing which program to implement; and (c) how to evaluate the impact of the program on clients served. Each subsection is further organized into a series of more detailed questions to guide CBOs in the program selection and implementation process.

Several unique features are integrated into the Toolkit. Narrative text, lists, diagrams, and tables serve as a guide for community organizations through the process of selecting, implementing, and evaluating EBPs. Throughout the Toolkit, links have also been provided to useful materials from other organizations. Additionally, the Toolkit features an interactive flowchart that helps organizations estimate and increase their readiness to implement EBPs, regardless of their previous experience.

A section about sustaining the implemented program is also included. It acknowledges a major element, the difficulty of ensuring that the implemented program is maintained in the face of changes in funding, resource availability, and audience characteristics. The Toolkit offers strategies that can help organizations increase the sustainability of their implemented program.

## What are Plans for Future Development?

EvidenceToPrograms.com was developed and is maintained as part of the CRC-Senior Health’s mission to engage individuals and their communities in programs that improve senior health and well-being. It is a critical element in a greater movement to improve population health via the availability and accessibility of community-based health supports and programs. Evidence-based programming provides CBOs an opportunity to take the lead in health promotion and to support seniors in self-management of chronic health conditions in settings that are associated with health, wellness, and leisure.

The CRC-Senior Health is dedicated to further development of EvidenceToProgram.com and is receptive to recommendations from Toolkit users as well as policy makers in the evidence-based programming arena. Broader use and dissemination of the Toolkit will allow CBOs throughout the US to improve the selection, implementation, and evaluation of EBPs, thus, enabling CBOs to effectively access and implement programs that match their clients’ needs.

## Conflict of Interest Statement

The authors declare that the research was conducted in the absence of any commercial or financial relationships that could be construed as a potential conflict of interest.

This paper is included in the Research Topic, “Evidence-Based Programming for Older Adults.” This Research Topic received partial funding from multiple government and private organizations/agencies; however, the views, findings, and conclusions in these articles are those of the authors and do not necessarily represent the official position of these organizations/agencies. All papers published in the Research Topic received peer review from members of the Frontiers in Public Health (Public Health Education and Promotion section) panel of Review Editors. Because this Research Topic represents work closely associated with a nationwide evidence-based movement in the US, many of the authors and/or Review Editors may have worked together previously in some fashion. Review Editors were purposively selected based on their expertise with evaluation and/or evidence-based programming for older adults. Review Editors were independent of named authors on any given article published in this volume.

## Footnotes

^1^The Community Research Center for Senior Health is a multi-institutional, multi-disciplinary research center created to develop, implement, evaluate, and disseminate evidence-based interventions that address multiple social and behavioral determinants of senior health (http://seniorhealth.sw.org).

